# Fairness and Eligibility to Long-Term Care: An Analysis of the Factors Driving Inequality and Inequity in the Use of Home Care for Older Europeans

**DOI:** 10.3390/ijerph14101224

**Published:** 2017-10-14

**Authors:** Stefania Ilinca, Ricardo Rodrigues, Andrea E. Schmidt

**Affiliations:** 1 European Centre for Social Welfare Policy and Research, 1090 Vienna, Austria; rodrigues@euro.centre.org; 2 Austrian Public Health Institute, 1010 Vienna, Austria; Andrea.Schmidt@goeg.at

**Keywords:** long-term care, home care services, inequality, decomposition, equity in use

## Abstract

In contrast with the case of health care, distributional fairness of long-term care (LTC) services in Europe has received limited attention. Given the increased relevance of LTC in the social policy agenda it is timely to evaluate the evidence on inequality and horizontal inequity by socio-economic status (SES) in the use of LTC and to identify the socio-economic factors that drive them. We address both aspects and reflect on the sensitivity of inequity estimates to adopting different definitions of legitimate drivers of care need. Using Survey of Health, Ageing and Retirement in Europe (SHARE)data collected in 2013, we analyse differences in home care utilization between community-dwelling Europeans in nine countries. We present concentration indexes and horizontal inequity indexes for each country and results from a decomposition analysis across income, care needs, household structures, education achievement and regional characteristics. We find pro-poor inequality in home care utilization but little evidence of inequity when accounting for differential care needs. Household characteristics are an important contributor to inequality, while education and geographic locations hold less explanatory power. We discuss the findings in light of the normative assumptions surrounding different definitions of need in LTC and the possible regressive implications of policies that make household structures an eligibility criterion to access services.

## 1. Introduction

As a growing number of older people in Europe find themselves in need of support, the policy relevance of long-term care (LTC) organization has considerably increased [1]. Despite the attention afforded to the increasing share of frail older people and the high costs associated with it, two aspects have been mostly overlooked in the literature: the issue of how equitable the use of LTC is and how this might vary between countries in Europe [2,3]. This stands in contrast with health care, for which a wide body of literature attesting to a socio-economic gradient in the use of different forms of health care has accumulated [4,5,6,7]. The gap is all the more remarkable as LTC in Europe is characterized by a wider diversity in terms of both allocation of public resources and the breadth and depth of coverage than health care [8,9]. The present study contributes to addressing this shortcoming by exploring the existence of inequalities in the use of LTC among community-dwelling older people in Europe and by comparing the results between European countries. Two main research questions are addressed. Firstly, what is the evidence of inequality and horizontal inequity by socio-economic status (SES) in the use of LTC at home across European countries. Secondly, which socio-economic factors drive inequality and inequity in use of LTC in different European countries. In addressing the second research question, we discuss the categorization of different factors driving inequalities into legitimate or illegitimate sources of variation in LTC use. We find that home-based care utilization is higher among lower income individuals in most European countries and that health needs and the structure of the household are its main drivers. Differences in use are almost entirely explained by differential care needs—i.e., we do not find evidence of horizontal inequity, but these results are sensitive to the categorization of household structures as legitimate rather than illegitimate sources of variation.

Previous work on LTC use has usually assumed that because older people of lower SES have on average poorer health [10], they would be the most likely users of LTC services. There is, however, a dearth of empirical evidence attesting to this situation and it is often limited to single country studies. What is more, the disparate results do not provide a consensual picture. Paraponaris and colleagues [11] report a socio-economic gradient in the use of formal care in France that persists even after controlling for differences in health. In The Netherlands, Geerlings and colleagues [12] produce a more nuanced picture of the socio-economic gradient, reporting that higher income was positively associated with the use of privately paid home care, but not with subsidized home care. Hoeck and colleagues [13] find that frailty is a strong predictor of LTC use among older Belgians and that it associates with socio-economic status, but their findings are inconclusive as to whether SES alone is a factor in LTC use. For Spain, studies offer contradictory evidence of the existence of a socio-economic gradient in the use of LTC services favouring the more affluent [14,15]. In Sweden, Larsson and Silverstein [16] find evidence for a socio-economic gradient, while Meinow and colleagues [17] do not. Schmidt [18] reports evidence of pro-rich inequity in the use of home care, based on administrative data for older urban dwellers in Austria, although mediated by household composition and gender. Comparative studies have been limited to few countries and the evidence of a socio-economic gradient in the use of LTC services after controlling for health is not entirely conclusive. Motel-Klingebiel and colleagues [19] report no differences in use of LTC services by SES for any of the countries studied: Norway, England, Spain, Israel and Germany. Van Groenou and colleagues [20] also report no differences in LTC use by income for Great Britain, The Netherlands and Belgium, while in Italy they find inequality favouring the poorer among older people. In a comparative study on Italy and Germany, Albertini and Pavolini [3] show a positive correlation between SES and use of LTC services, which is absent in France and Denmark. Finally, Bakx et al. [2], comparing Germany and The Netherlands, report inequalities in the use of LTC services for the former but not for the latter, even after controlling for confounding differences in health and disability.

Other socio-economic factors may also impact inequalities in the use of LTC services. Chiefly among them are household structures (e.g., marital status, number of children and frequency of contacts with them, cohabitation) as they may determine access to informal care or ability to count on potential ‘advocates’ to receive LTC services [16,21,22,23,24]. Results from social network studies indicate that higher socio-economic condition correlates positively with greater household size and closer distance to children, pointing to an advantage to receive informal care among higher income groups [25]. Concomitantly, the quality of social contacts was found to be higher among the middle class, with existing contacts translating into personal support more easily than among older people in working classes [26]. As a counterweight to this, filial obligations seem to be stronger among lower socio-economic groups [27,28] and findings from the informal care literature point to a higher probability of receiving care among lower SES groups [29]. Household structure may thus mediate socio-economic differences in the use of LTC services, as shown by Schmidt [18], who found a very different socio-economic gradient between singles (pro-rich) and non-singles (inexistent) in the use of LTC services.

Another potential source of inequalities stems from the leeway that regional or local governments have in determining eligibility criteria and availability of LTC [30]. More affluent regions or municipalities may provide more services (i.e., have lower eligibility thresholds) or be able to attract more providers and this postal code lottery may be exacerbated by older people’s limitations to travel or relocate. On this matter, available empirical findings suggest plenty of geographical variation across countries [30,31,32,33,34], but these seem to be small and closely associated with geographical distribution of need and may not therefore reflect inequity.

The existing literature is therefore not always conclusive as to the existence of inequalities and inequities in the use of LTC by socio-economic status in Europe and as to possible factors associated with those inequalities. Furthermore, there is still a lack of clarity with respect to which factors should be considered sources of legitimate or ‘fair’ variation, and which may be considered sources of illegitimate or ‘unfair’ variation in analyses of equity in access to care [35]. While the health endowment of individuals is unequivocally a need-driven factor, income-related differences as well as differences related to other social background factors (like education, and living in an urban area) are considered illegitimate factors. It is less clear how to treat household structure. Some theorists have argued that personal preferences are shaped by an individual’s experience over the life course, and these are in turn influenced to a large degree by resource availability, i.e., socio-economic status [36,37,38]. Therefore, indicators of household structure might be considered as non-need factors that represent illegitimate sources of variation in LTC use. At the same time, some empirical studies have shown that living alone in older age is a difficult and detrimental condition which can hardly be compensated by the use of formal LTC services alone [16,21,39]. From this perspective, household structures (especially the absence of any informal support) might be considered as requiring particular attention in the allocation of public LTC services for older people, and therefore as legitimate variations in LTC use. Among the countries in our sample, only in The Netherlands is the composition of the household (i.e., the presence of able spouses in the household) explicitly recognized as an eligibility criterion to LTC [2], but the introduction of similar measures has been discussed in policy fora in many other contexts.

Using methods commonly employed in the study of inequalities in health care, in the following, we attempt to systematically explore the main factors that contribute to income-related inequality and inequity in the use of home care among community-dwelling older people in Europe. The analysis carried out in this study covers nine European countries, allowing us to present a comprehensive picture of inequalities and inequities in LTC use. The analytical strategy employed is two-fold. Firstly, for each country in the sample, socio-economic inequality in use of home care services is explored by estimating concentration indexes and identifying the factors most strongly associated with income-related inequality in use via decomposition analysis. Secondly, we explore whether income-related differences remain after controlling for possible systematic differences in the health condition of individuals. In this second part of the study we also explore the impact of using different definitions of need variables on the observed inequity in the use of home care in Europe, particularly household composition and structure, the most important non-health related drivers of inequality.

## 2. Materials and Methods

In assessing socio-economic inequality in access to LTC we follow a well-established literature in health care and employ the Concentration Index (CI) [40,41]. The CI is a synthetic measure of socio-economic related health inequality, bounded between −1 and 1. It takes negative values when the long-term care use variable is disproportionately concentrated among the poor and positive values when inequality favours the rich. It can be calculated via the “convenient regression” expression as:

(1) $$CI=\frac{2}{\mu }cov\left(h_{i},r_{i}\right)$$

where, $h_{i}$ is the care utilization variable, μ is its average and $r_{i}$ denotes the fractional rank of each individual in the SES distribution. Because the care utilization variable we employ is bounded, a scale correction is necessary [42], resulting in the corrected concentration index (*CCI*):

(2) $$CCI=4\times \mu \times CI=8cov\left(h_{i},r_{i}\right)$$

While the concentration index offers an overview measure of total socio-economic inequality, it offers no insights into the factors that determine the degree of inequality observed, a question of particular relevance for policy-making. Such a decomposition into the contributions of different explanatory factors is possible for the *CI* via a regression analysis technique proposed by Wagstaff and colleagues [43]. If we start from an explanatory model, such as:

(3) $$h_{i}=\alpha +\underset{k}{\sum }\beta_{k}x_{ki}+\varepsilon_{i}$$

where $x_{k}$ is a vector of variables associated with care utilization and ε is the error term, then we can rewrite the *CI* as:

(4) CI=∑k(βkx¯kh¯)CIk+GCεh¯

where ${\bar{x}}_{k}$ is the mean of $x_{k}$, ${CI}_{k}$ is the concentration index for regressor $x_{k}$ and ${GC}_{\varepsilon }$ is the generalized concentration index of the error term. In this specification, the CI is expressed as the sum of the contributions of all considered factors (the $x_{k}$ vector) and an error component (called the generalized concentration index for the error term). For non-linear models, comparable results can be obtained from a linear approximation of the model based on a partial effects representation [4].

It also becomes apparent from the formulation in Equation (4) that the magnitude of the contribution of each considered factor will depend on two measures: (i) how sensitive long-term care use is to variation in the given factor—i.e., its elasticity with respect to it (βkx¯kh¯); and (ii) how equal is the distribution with respect to income of the given factors—i.e., its concentration index $CI_{k}$ [44]. It is the factors that are both unequally distributed and associated with long-term care use that will have the largest contributions to overall inequality. One can consider as an example the case of education achievement. Even if higher education is likely to be concentrated among the more affluent individuals, its contribution to the overall *CI* for home care utilization will be negligible unless the level of education is also a strong predictor of care utilization. Similarly, as the contribution of each factor is calculated as the product of its concentration index and the elasticity of long-term care use with respect to it, the sign each takes will play a role in determining whether the factor contribution favours the poor (negative sign) or rather, the richer individuals in the sample. Finally, as total inequality in use is calculated as a sum over the contributions of all considered factors, it is also important to consider that factor contributions can either reinforce each other (i.e., same sign) or cancel each other out (opposite sign).

While analyses of inequality focus exclusively on the distribution of care utilization, it is often of more interest for both policy makers and researchers to understand if and to what extent care utilization is driven by dependency and need for care—an analysis of equity. The horizontal inequity (HI) index reflects the homonymous principle that individuals with equal care needs should receive equal amounts of care and is a measure of the difference between actual and need-predicted care utilization. The HI is derived through the indirect standardization method [41]. Starting with the vector of determinants of LTC utilization $x_{k}$ and separating the determinants of care use into a need and a non-need vector, one can use a logistic regression model to estimate how much care each individual would have received, had she been treated in the same way as were, on average, other individuals with equal care needs. Formally, if we assume $h_{i}$ to be a linear and additively separate function of need ($N_{k}$) and non-need indicators ($Z_{j}$), as follows:

(5) $$h_{i}=\alpha +\underset{k}{\sum }\beta_{k}N_{ik}+\underset{j}{\sum }\delta_{j}Z_{ij}+\varepsilon_{i}$$

the CI can be written as the weighted average of the CIs for all included indicators [45] and the HI (horizontal inequity index) can be obtained as the difference between the CI for need-predicted utilization and the CI for actual (observed) utilization. Similarly to the CI, values below zero for the HI indicate a pro-poor distribution of the use variable, while positive values indicate pro-rich inequity.

The analysis is based on micro-data from the fifth wave of the Survey of Health, Ageing and Retirement in Europe (SHARE), collected during 2013 in nine European countries—Austria, Germany, Sweden, The Netherlands, Spain, Italy, France, Denmark and Belgium [46,47]. We maintained in the sample all individuals aged 60 or above, who personally or via a proxy have completed the questionnaire and whose long-term care utilization and socio-economic status could be identified in the data. Imputed values (provided in the most recent SHARE release) were used to replace missing values for all SES and control variables [48], in order to ensure that sample sizes are maximized whenever possible. The final analysis sample consists of 31,389 community-dwelling older individuals.

Long-term care use is measured by an indicator of formal care services utilization in the home during the 12 months before the interview that captures professional or paid-for support including personal care, domestic tasks, other activities and meals on wheels. Socio-economic status is proxied by equivalized net household income, obtained via the household level aggregation of all income components (including social benefits), equivalized using the square root scale [49] and adjusted for purchasing power parity. Sample sizes by country and descriptive statistics for all the variables used in the analysis are presented in Table 1. Need variables include less than good self-rated health, presence of moderate or severe disability (indicated by activities of daily living (ADL) limitations), number of diagnosed chronic illnesses, poor mental health status, presence of long-term illness diagnosed by a physician, frailty, age and gender. Non-need factors are education achievement (primary, secondary or tertiary), the region of residence (Nomenclature of Territorial Units for Statistics (NUTS) 2 level) and the level or urbanization. Because the reference region is always selected to be the region containing the country capital, our regional characteristics factor can be interpreted to reflect differences in population density and urbanization. Finally, household characteristics, treated in turn as both need and non-need factors, include the reported size of the household, the number of children of the respondent (that may or may not reside in the same household) and the marital status.

## 3. Results

### 3.1. Descriptive Statistics

The results of our analysis confirm the high variability between population and care system characteristics across the European landscape (see Table 1). It is noteworthy that the share of people using home care services varies widely, from under 10% of the population aged 60 years and older in Sweden and Italy to as much as one fifth (23%) in Belgium and over 17% in France. Income inequality among households led by older individuals, as measured by the Gini coefficient, is highest in France, Belgium and Italy, while Austria, Denmark and Sweden are the countries with the least unequal income distribution. Most individuals considered in the sample have attained at least secondary education, with Spain (approximately 33%) and Italy (45%) being the only two exceptions.

Regarding household structure and family composition, Spain and Italy are the countries where the average household size tends to be largest, reflecting a higher prevalence of intergenerational cohabitation. The average number of children of people aged 60 years and older varies from 2.02 in Germany to 2.42 in Spain. In all countries included in the sample, most older people are married, albeit substantial differences are observed: in Austria and France, approximately 63% are married, whereas in The Netherlands, Spain and Italy almost 80% of those aged 60 years and older are married.

### 3.2. Analysis of Inequality in Access to Home Care

Overall, the results of our analysis show that in most countries considered in our sample, home care services are disproportionately concentrated among the poorest people in the older population, as indicated by the CI values listed in Table 2. Exceptions to this pattern are Spain and Italy, for which CIs are not statistically significant from zero. In contrast, the highest pro-poor inequalities are present in Denmark, The Netherlands and France.

**Figure 1:**
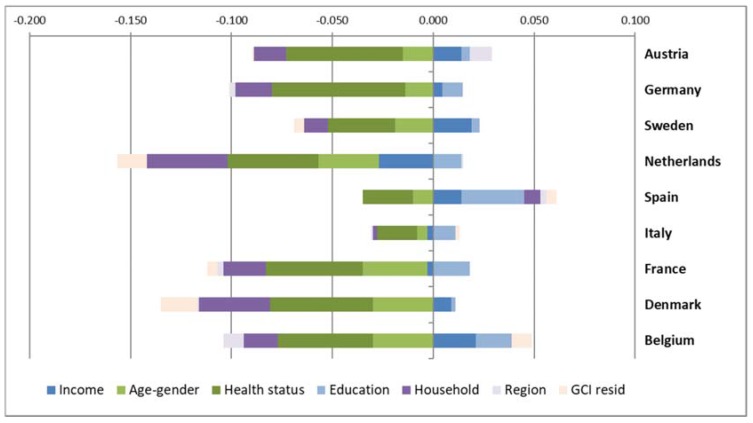
Decomposition of CIsin home care utilization.

A better understanding of the dynamics driving inequality in the use of home care services is afforded by the decomposition analysis (see Figure 1), which breaks down the overall CI value in the individual contributions of each need and non-need factor considered. This synthetic visualization of results highlights that care needs, proxied by health status and socio-demographic characteristics (dark and light green bars), together with household characteristics (dark purple bars), are the main driving factors of inequalities in use of home care services across the nine European countries we consider, i.e., they contribute the most in relative terms to the value of the CI of each country. In contrast, we find that regional characteristics and education achievement play more contained roles.

As the bars span both right and left from the origin point, they indicate that the contributions of different factors can pull in opposite directions. Thus, the contribution of poor health drives pro-poor inequalities (negative sign). With the noteworthy exception of Spain, poor health status is the factor with the highest impact on pro-poor inequality in use of home care, closely followed by (older) age and (female) gender. As these are also the factors considered to reflect legitimate needs, the decomposition results offer a first hint into why the concentration indexes are negative for all countries in our sample: because care needs concentrate disproportionately among lower income individuals. This can be verified with the detailed decomposition results presented in Table 3. All the poor health indicators we consider in the analysis are more common among lower income individuals (negative CI values are registered for all indicators and for all countries in our sample). Furthermore, they are all positively associated with home care utilization. We observe only two exceptions where the association with income is positive: presence of chronic conditions in Italy and poor mental health in The Netherlands. Poor self-rated health, the presence of severe ADL limitations and of chronic conditions make the largest contribution to pro-poor inequality, due to larger positive elasticities of home care use to these factors and, in the case of severe ADL limitations, also due to its high concentration among lower income groups. Conversely, poor mental health and cognitive impairment, while being highly concentrated among the poor make relatively smaller contributions to inequality due to elasticity values closer to zero.

Household characteristics also contribute to pro-poor inequalities in use of home care in virtually all the countries analysed. This is the case because larger households and being married tend to be concentrated among richer individuals (positive CI values) while being associated with reductions in the use of formal home care (negative elasticity), thus leading to negative contributions (the product of the two) to overall inequality in use (see Table 3). The exception is the number of children, since this indicator is also inversely associated with home care use (negative elasticity) but, unlike household size and marital status, is concentrated among lower income families (negative CI). The balance between the positive and the negative contributions of the variables considered under household structures is generally positive because both the elasticities and the CIs for the number of children are relatively smaller than those observed for household size and being married.

Exceptionally, in Spain, the contribution of household structures is positive (pro-poor), mostly due to the fact that larger households are concentrated among poorer individuals (negative CI). This is unusual in our sample of countries, with only Italy and Austria paralleling the pattern found in Spain. The highest overall contributions of household characteristics are observed in The Netherlands and in Denmark, with Italy at the opposite pole (mostly due to a very low contribution of household size to overall inequality levels).

The socio-economic characteristics of home care users, measured as education achievement and household income, generally contribute to pro-rich inequality. One notable exception to this pattern are the negative contributions of income in the cases of France, Italy and most markedly, The Netherlands. Counterintuitively, these results seem to suggest that higher incomes are associated with lower access to home care services. In fact, the negative sign of the contribution of income in all three instances is driven by the negative elasticity of home care utilization to changes in income (i.e., larger income associated with lower home care utilization) while the CI is positive (by definition, higher incomes are concentrated in richer groups). In the case of education, it is the uniform and very high concentration of tertiary education achievement among higher income individuals that plays the decisive role in driving the overall positive contribution of this factor. In contrast, secondary education is more frequent among poorer individuals in five out of nine countries in our sample while in four cases elasticities are negative.

Our results show a very limited contribution of regional characteristics in all countries. This is to a large extent the effect of contrasting contributions from different indicators (mostly regional dummies) within this category, cancelling each other out (results are available from the authors on demand). Nonetheless, the results for our indicators of urbaneness paint an interesting picture. While lower income individuals are more likely to live in rural environments, the latter variable is not necessarily associated with lower access to care services. In fact, in five countries in our sample (Belgium, Denmark, France, Germany and Sweden) the elasticity of home care use to living in a rural environment is positive, indicating that service coverage in lower population density areas is not under-developed.

### 3.3. Inequity Analysis

The results of the decomposition analysis highlighted the contribution of health and dependency status indicators—i.e., *needs* variables—to the observed income-related inequality in home care utilization. As these variables can unequivocally be categorized as legitimate sources of variation in use between different income groups we estimated horizontal inequity (HI) indices, in order to ascertain whether differences between groups remain after accounting for differences in care needs. However, the decomposition analysis also revealed the very large contribution to overall inequality of household structures with the potential to affect the robustness of results depending on their categorization into the need rather than the non-need category. However, to the best of our knowledge, no explicit discussion on the topic that accounts for the particularities of LTC provision is available in the literature and grounding in the theoretical or empirical literature on inequity in care utilization provides insufficient guidance. Furthermore, while it is more common in current LTC policies in Europe not to consider household composition in establishing eligibility to care, this norm is increasingly being called into question in the policy debate. Therefore, rather than a-priori taking a normative stance on the legitimacy of household structure factors in driving LTC use, we opted to run two parallel analyses (see Table 4) that are identical in every way other than the identification of indicators for household structures as non-need respectively need factors.

Firstly, household characteristics (including marital status, household size and number of children) are treated as non-need factors, i.e., illegitimate as they do not drive a valid reduction in the need for care. In this model, results are generally not statistically significant, with three exceptions. In two countries, negative HI coefficients are observed, namely in Denmark (HI = −0.063) and The Netherlands (HI = −0.040), indicating a significant pro-poor orientation of home care services, even after taking into account the higher care needs in this group. The only country where significant pro-rich inequity is found, as indicated by the positive HI coefficient, is Spain (HI = 0.067). These HI results, in noticeable contrast with the values of the CIs for home care utilization, strongly indicate that differences in care utilization are largely explained by differences in care needs between richer and poorer groups of older individuals. They do not reflect, as the inequality analysis would have suggested, an effective pro-poor targeting of home care services, in most European countries.

In the second part of the inequity analysis, household characteristics are considered to be ‘fair’—or needs-related—sources of inequality in the distribution of home care services (see Table 4, columns 5 and 6), rooted for instance in personal circumstances that may not be altered by personal choices alone. Among the countries in our sample, this is effectively the case only in The Netherlands, where people without informal carers are entitled to a higher amount of LTC services However, the exercise is informative in that it helps provide a glimpse into the effect on equity in access to care, should often discussed policy reforms be adopted in other European countries considered here. Once household factors are considered part of the needs vector and a legitimate source of inequality, the HI coefficients in virtually all countries indicate higher pro-rich inequity, albeit Spain remains the noteworthy exception. In Germany and Sweden, previously pro-poor inequity becomes pro-rich inequity (sign change) albeit none of the HIs are statistically significant, while in Denmark, a previously strongly significant pro-poor HI loses statistical significance. Furthermore, previously not statistically significant but positive HI coefficients for Austria, Italy, France and Belgium now display statistical significance levels of inequity in access to home care services utilization. Overall, in six out of nine countries analysed, the choice of whether to treat household characteristics as need or non-need factors leads to a change in either the sign or the significance level of the HI and therefore a change in the equity conclusion on the allocation of home care services to older users in different income groups.

## 4. Discussion

The results presented in this paper make a noteworthy contribution to the emerging literature on equity in the use of long-term care in three important ways. Firstly, they contribute to building an evidence base that long-term care utilization varies not only between countries but also between income groups within countries and that this variability persists even after differences in need are controlled for. Secondly, they provide novel and important insights into the factors that drive such inequalities and their distribution across income groups. Thirdly, our findings highlight that estimates of inequity in the use of LTC are sensitive to the used definition of need factors. This is particularly relevant given the lack of an explicit definition or empirical consensus on what constitutes fair and unfair sources of inequality, especially in relation to the availability of informal care support (proxied by household characteristics). We discuss each of these findings in turn.

Using recent data from a comparable cross-national survey, the findings presented here confirmed large disparities between the levels of formal care utilization between different European countries [50,51]. Such differences are likely to be driven by variations in the organization and generosity of LTC systems throughout Europe [2,3], but also by differences in the prevalence of need. The comparatively low use of home care in Sweden relative to other countries, could be partly explained by the fact that only approximately 8% of older Swedes report limitations in ADLs; in comparison, this share is twice as high in Belgium. While most countries in our sample displayed a socio-economic gradient in the use of home care services favouring the poor, most of these differences reflect dissimilar care needs across income groups. After adjusting for higher needs in lower income groups, we found that only Denmark and The Netherlands successfully target home care services to poorer individuals. Confirming previous results, Spain, a country that relies on means-testing for service targeting, stands out in comparison with other Western European countries, with a clear pro-rich distribution of home care services [14]. Given the prevalence of needs-based eligibility criteria for access to home care among the countries studied [52], these results show a relative under-achievement with respect to the distributional fairness of home care services in Europe.

The second important contribution of our study is the identification of the main factors driving inequality in use of home care in Europe. This is, to the best of our knowledge, the first study to produce a decomposition of concentration indices for long-term care utilization and therefore offers novel insights into possible causes for inequality in the use of services. In the first instance, inequalities are driven by different prevalence rates of disability, poor health and frailty across income groups, confirming results of previous studies on health and disability gradients in health among older people [53,54]. Almost as important in explaining unequal use of services are household characteristics. This is in line with a wide body of literature that has shown informal care use to be concentrated among less affluent individuals [14,20,29].

Thirdly, our study draws attention to the fact that there is no commonly agreed upon definition of what can be considered as justified variation in home care use, particularly as it relates to the treatment of indicators of social and informal care support. This pervasive lack of clarity in the normative separation of fair from unfair inequalities means that applied analyses of equity in long-term care could easily reach diametrically opposed conclusions, depending on the assumptions and choices the analyst makes with respect to the treatment of household characteristics. In this respect, international experience and practice offers no guidance as carer-blind and carer-sensitive systems currently coexist and have historically coexisted in Europe. What seems, however, clear from our results is that the choice to condition the use of formal long-term care services on the availability of informal care will disproportionately affect lower income families and will lead to long-term care systems significantly biased in favour of the rich. As their consequences for equity achievement in long-term care systems are sizeable, we encourage policy makers to open a broad dialogue on these issues and urge researchers to further explore them in studies that cover a wider sample of countries and that reflect on other types of formal care services.

Finally, we wish to acknowledge some limitations of our study that stem from the data and methodological approach employed. While our results are based on a rich and comparable cross-country dataset, some issues can be raised with the quality of data. We rely exclusively on self-reported measures of both utilization and health and socio-economic status, without any possibility to validate them against objectively measured or administrative data. In addition, we acknowledge that not all dimensions considered in our analysis are measured via equally precise and satisfactory indicators. A case in point is the measurement of regional differences via an indicator on NUTS2 statistical regions. Further research, based on finer grained geographical data, should focus on testing the existence of spatial inequalities in use of LTC. In a similar vein, as our results rest on cross-sectional data and could reflect time or sample idiosyncrasies, future longitudinal studies are needed in order to confirm their robustness. Lastly, our analysis is limited in that it is focused on the use of home-based care services, without controlling for the availability and use of alternative care types. Most noteworthy among these alternatives, albeit for different reasons, are the amount of informal care received and the number of people in institutional care. In the former case this is relevant because informal care received in the home setting can act as a substitute to formal long-term care use and thus have implications for the distribution of the use of home care services [50,55,56]. In the case of institutional care, dissimilar probabilities to be institutionalized by income might introduce bias in the measurement of inequalities (e.g., if people with low socio-economic status disproportionately enter institutional care). Therefore, we encourage future studies that can link available administrative and survey-based data in order to evaluate inequality and inequity in access to different types of long-term and health care services concomitantly.

## 5. Conclusions

Our results contribute to a growing, but as of yet under-developed area of research on inequality and distributional fairness of formal long-term care services in Europe. While poorer individuals are the main users of home care services, most countries seem to under-perform with respect to the goal of reaching horizontal equity in home care services (i.e., equal treatment for equal needs). This is cause for severe concern in light of current demographic and social trends and highlights the need for care policies to focus on equity rather than equality in access to care. Furthermore, because equality and equity in use are conditioned on more than simply providing a levelled playing field by equalizing the starting point for LTC use, policies focused exclusively on supply side factors are bound to improve accessibility but only marginally impact utilization. Therefore, we argue for complex interventions, spanning multiple policy areas that address the multifaceted interactions between socio-economic factors and LTC use among older Europeans. Key insights for policy formulation are offered by the decomposition analysis, which points to the distribution of socio-economic characteristics that drive or hamper care utilization between different income groups.

Furthermore, our study shows that the way formal care systems treat the availability of informal care support is a non-trivial matter and can lead to marked changes in equity conclusions. This is, ultimately, a normative question and we believe one that needs to be discussed and addressed with urgency as policy measures that limit access to publicly funded services based on the availability of informal care support are regularly and increasingly discussed and even implemented as reform alternatives for LTC systems in Europe. We hold that it is imperative for policy-makers to weigh aspects related to costs and sustainability with distributional and fairness concerns. Namely, we caution that shifting the responsibility and burden of care from the formal sector onto family and informal caregivers will preponderantly affect lower income households and disproportionately favour the rich.

## Figures and Tables

**Table 1:** Descriptive statistics for dependent and independent variables by country, percent/mean (standard deviation).

	AT	DE	SE	NL	ES	IT	FR	DK	BE
Age (in years), 60+	70.99	70.40	71.13	70.28	72.90	71.41	71.74	70.63	71.25
	(7.70)	(7.62)	(7.73)	(7.64)	(8.83)	(7.72)	(8.67)	(7.96)	(8.34)
Female (%)	56.63	50.34	51.85	52.67	52.66	52.39	56.38	52.14	53.58
	(49.56)	(50.00)	(49.97)	(49.93)	(49.93)	(49.94)	(49.60)	(49.96)	(49.88)
Less than good self-rated health (%)	35.33	44.40	26.11	31.38	48.89	49.78	41.23	24.55	30.45
	(47.81)	(49.69)	(43.93)	(46.41)	(49.99)	(50.00)	(49.23)	(43.05)	(46.02)
Has moderate ADL limitations (%)	7.91	8.17	6.50	5.75	7.87	9.63	11.10	7.43	13.85
	(26.9)	(27.40)	(24.66)	(23.29)	(26.93)	(29.51)	(31.42)	(26.23)	(34.55)
Has severe ADL limitations (%)	3.95	3.84	1.74	1.48	7.56	5.77	3.39	2.07	3.99
	(19.50)	(19.22)	(13.05)	(12.11)	(26.44)	(23.33)	(18.11)	(14.24)	(19.58)
Number. of chronic illnesses	1.91	2.25	1.97	1.83	2.24	2.04	2.01	2.05	2.09
	(1.17)	(1.34)	(1.20)	(1.15)	(1.31)	(1.27)	(1.20)	(1.23)	(1.25)
Has long-term illness (%)	50.87	62.82	53.07	51.64	53.30	46.54	49.80	52.39	49.83
	(50.00)	(48.34)	(49.91)	(49.98)	(49.89)	(49.89)	(50.00)	(49.95)	(50.00)
Has poor mental health status (%)	19.64	23.04	17.99	16.35	33.39	37.88	34.93	16.24	27.95
	(39.73)	(42.11)	(38.41)	(36.98)	(47.16)	(48.51)	(47.68)	(36.89)	(44.88)
Has cognitive impairment (%)	2.93	1.61	1.35	1.15	4.65	2.47	1.31	0.69	1.98
	(16.86)	(12.57)	(11.56)	(10.66)	(21.05)	(15.51)	(11.39)	(8.28)	(13.94)
Is frail (%)	34.20	28.52	33.48	32.87	40.66	34.19	49.54	29.19	43.94
	(47.44)	(45.15)	(47.19)	(46.98)	(49.13)	(47.44)	(50.00)	(45.47)	(49.64)
Completed secondary education (%)	60.43	69.16	46.05	61.71	24.67	38.20	39.43	48.27	48.84
	(48.90)	(46.18)	(49.85)	(48.61)	(43.11)	(48.59)	(48.88)	(49.98)	(49.99)
Completed tertiary education (%)	25.25	28.98	27.85	25.66	8.15	6.82	19.18	37.66	30.17
	(43.45)	(45.37)	(44.83)	(43.68)	(27.37)	(25.21)	(39.37)	(48.46)	(45.91)
Lives in a town (%)	20.91	34.54	52.73	42.98	69.51	42.01	35.64	48.62	47.64
	(40.67)	(47.56)	(49.93)	(49.51)	(46.04)	(49.37)	(47.90)	(49.99)	(49.95)
Lives in rural area (%)	44.94	41.98	17.70	22.26	6.64	37.76	47.17	22.83	27.28
	(49.75)	(49.35)	(38.17)	(41.60)	(24.90)	(48.48)	(49.93)	(41.98)	(44.55)
Number of household members	1.88	1.91	1.81	1.85	2.22	2.23	1.80	1.78	1.85
	(0.88)	(0.59)	(0.53)	(0.54)	(0.91)	(0.95)	(0.68)	(0.55)	(0.70)
Number of children	2.16	2.02	2.35	2.39	2.42	2.06	2.29	2.30	2.22
	(1.46)	(1.29)	(1.31)	(1.38)	(1.52)	(1.34)	(1.48)	(1.26)	(1.49)
Is married (%)	63.36	75.92	73.37	78.06	77.74	76.78	63.26	70.00	68.39
	(48.19)	(42.76)	(44.21)	(41.38)	(41.60)	(42.23)	(48.21)	(45.83)	(46.50)
Use home care (%)	13.42	11.74	7.72	14.69	11.95	8.38	17.13	11.45	23.02
	(34.09)	(32.19)	(26.70)	(35.41)	(32.44)	(27.73)	(37.68)	(31.85)	(42.10)
Gini coefficient for household income	0.285	0.326	0.280	0.302	0.338	0.420	0.456	0.275	0.446
	(0.003)	(0.003)	(0.002)	(0.007)	(0.003)	(0.012)	(0.057)	(0.003)	(0.008)
***N***	**3212**	**3671**	**3691**	**2954**	**4905**	**3445**	**3269**	**2610**	**3632**

Note: AT—Austria, DE—Germany, SE—Sweden, NL—Netherlands, ES—Spain, IT—Italy, FR—France, DK—Denmark, BE—Belgium; ADL—activities of daily living; N—number of observations.

**Table 2:** Inequality in use of home care services, standard errors in parentheses.

	AT	DE	SE	NL	ES	IT	FR	DK	BE
**Concentration Index (CI)**	−0.078 ***	−0.088 ***	−0.080 ***	−0.161 ***	0.027	−0.009	−0.105 ***	−0.186 ***	−0.046 **
	(0.018)	(0.017)	(0.014)	(0.018)	(0.017)	(0.014)	(0.017)	(0.017)	(0.018)

Note: *** *p* < 0.001; ** *p* < 0.01.

**Table 3:** Decomposition results (selected need factors).

	**Self-Rated Health**		**Moderate Limitations**		**Severe Limitations**		**Chronic Conditions**		**Long-Term Illness**		**Mental Health**		**Cognitive Impairment**		**Frailty**	
	**Elast.**	**CI**	**Elast.**	**CI**	**Elast.**	**CI**	**Elast.**	**CI**	**Elast.**	**CI**	**Elast.**	**CI**	**Elast.**	**CI**	**Elast.**	**CI**
Austria	0.121	−0.179	0.101	−0.157	0.139	−0.247	0.254	−0.025	0.050	−0.053	0.035	−0.148	0.018	−0.194	0.069	−0.132
Germany	0.121	−0.146	0.108	−0.182	0.204	−0.187	0.165	−0.055	0.090	−0.059	0.056	−0.149	0.008	−0.242	0.102	−0.113
Sweden	0.098	−0.198	0.058	−0.231	0.058	−0.298	0.086	−0.059	0.177	−0.067	0.019	−0.174	0.024	−0.165	0.112	−0.145
Netherlands	0.117	−0.135	0.087	−0.166	0.042	−0.232	0.133	−0.048	0.090	−0.032	−0.006	−0.148	0.006	−0.295	0.107	−0.115
Spain	0.118	−0.096	0.040	−0.052	0.134	−0.197	0.157	−0.029	0.093	−0.037	0.041	−0.133	0.002	−0.083	0.018	−0.1
Italy	0.097	−0.084	0.033	−0.151	0.068	−0.118	−0.008	−0.042	0.034	−0.051	0.050	−0.106	0.021	−0.155	0.200	−0.112
France	0.150	−0.139	0.063	−0.130	0.069	−0.209	0.054	−0.032	0.149	−0.066	0.035	−0.091	0.011	−0.063	0.145	−0.07
Denmark	0.089	−0.226	0.058	−0.164	0.071	−0.401	0.100	−0.073	0.104	−0.080	0.010	−0.136	0.005	−0.355	0.123	−0.18
Belgium	0.114	−0.113	0.082	−0.074	0.066	−0.225	0.115	−0.027	0.084	−0.040	0.010	−0.055	0.002	−0.086	0.094	−0.091
	**Income**		**Household Size**		**No. of Children**		**Married**		**Urban**		**Rural**		**Secondary Education**		**Tertiary Education**	
	**Elast.**		**Elast.**	**CI**	**Elast.**	**CI**	**Elast.**	**CI**	**Elast.**	**CI**	**Elast.**	**CI**	**Elast.**	**CI**	**Elast.**	**CI**
Austria	0.732		−0.107	−0.005	0.016	−0.063	−0.209	0.132	−0.011	0.047	−0.162	−0.121	−0.014	−0.019	0.018	0.315
Germany	0.241		−0.150	0.042	−0.046	−0.038	−0.210	0.124	−0.005	0.024	0.066	−0.040	−0.214	−0.082	0.000	0.251
Sweden	1.621		−0.346	0.069	−0.063	0.024	−0.044	0.169	−0.013	−0.047	0.017	−0.015	−0.016	0.006	0.047	0.261
Netherlands	−1.072		−1.038	0.046	−0.038	−0.019	−0.071	0.121	−0.002	0.044	−0.046	−0.013	−0.071	−0.048	0.046	0.331
Spain	0.576		−0.461	−0.037	−0.124	−0.046	−0.080	0.058	−0.015	−0.052	−0.022	−0.058	0.089	0.148	0.109	0.523
Italy	−0.076		−0.173	−0.015	−0.103	−0.059	−0.180	0.072	−0.090	0.045	−0.083	−0.076	0.073	0.142	0.042	0.474
France	−0.090		−0.307	0.022	0.080	−0.018	−0.192	0.111	0.029	−0.019	0.015	−0.063	0.058	0.076	0.047	0.443
Denmark	0.591		−0.734	0.065	−0.135	−0.005	−0.140	0.145	0.064	−0.040	0.031	−0.009	0.009	−0.053	0.018	0.243
Belgium	0.506		−0.214	0.030	0.052	−0.015	−0.142	0.080	0.066	−0.001	0.041	−0.066	0.056	−0.039	0.088	0.243

**Table 4:** Horizontal inequity in the use of home care (with alternative specification of household structure role).

	Household Structures as Non-Need Factors		Household Structures as Need Factors	
Country	HI	Std. Error	HI	Std. Error
**Austria**	0.005	0.014	0.026 *	0.014
**Germany**	−0.005	0.013	0.012	0.013
**Sweden**	−0.012	0.012	0.007	0.012
**Netherlands**	−0.040 *	0.016	0.007	0.015
**Spain**	0.067 ***	0.015	0.063 ***	0.015
**Italy**	0.024	0.013	0.028 **	0.013
**France**	0.008	0.014	0.030 **	0.014
**Denmark**	−0.063 ***	0.014	−0.008	0.013
**Belgium**	0.020	0.016	0.037 **	0.016

Based on weighted data. Legend * *p* < 0.10; ** *p* < 0.05; *** *p* < 0.01.
